# Acidic Polysaccharide Extracts from *Gastrodia* Rhizomes Suppress the Atherosclerosis Risk Index through Inhibition of the Serum Cholesterol Composition in Sprague Dawley Rats Fed a High-Fat Diet

**DOI:** 10.3390/ijms13021620

**Published:** 2012-02-02

**Authors:** Kui-Jin Kim, Ok-Hwan Lee, Chan-Kyu Han, Young-Chan Kim, Hee-Do Hong

**Affiliations:** 1 Department of Cancer and Cell Biology, University of Cincinnati Medical Center, Cincinnati, OH 45267, USA; E-Mail:kimkj@ucmail.uc.edu; 2 Department of Food Science and Biotechnology, Kangwon National University, Chuncheon 200-701, Korea; E-Mail: loh99@kangwon.ac.kr; 3 Korea Food Research Institute, Seongnam, Kyonggi 463-746, Korea; E-Mails: ckhan@kfri.re.kr (C.-K.H.); yckim@kfri.re.kr (Y.-C.K.)

**Keywords:** *Gastrodia* rhizome, acidic polysaccharide, cardio vascular disease, atherogenic index

## Abstract

Obesity is associated with a broad spectrum of cardio-metabolic disturbances, including atherosclerosis and cardiovascular disease (CDV). A high-fat diet has been shown to cause an elevation of the plasma cholesterol levels in humans, and the control of serum cholesterol has been demonstrated to be important in the prevention of CVD and atherosclerosis. The aims of this study were to demonstrate that crude and acidic polysaccharide extracts from *Gastrodia* rhizomes suppress atherosclerosis through the regulation of serum lipids in Sprague Dawley (SD) rats fed a high-fat diet. We examined the concentrations of serum lipids, including total cholesterol, triglycerides, high-density lipoproteins (HDL) cholesterol, and low-density lipoproteins (LDL) cholesterol, in SD rats fed a high-fat diet and evaluated the atherogenic index. Here, we show that both crude and acidic polysaccharide extracts from *Gastrodia* rhizomes inhibited the total cholesterol and LDL levels. Moreover, there was a significantly suppressed atherosclerosis risk due to the acidic polysaccharide extract from *Gastrodia* rhizome. Taken together, our results suggested that acidic polysaccharide extracts from *Gastrodia* rhizomes might be beneficial for lowering the incidence of CVD and atherosclerosis by reducing the *de novo* synthesis of total cholesterol and the LDL levels.

## 1. Introduction

The prevalence of overweight and obese individuals worldwide has increased dramatically due, in large part, to the over consumption of a high-fat and high-cholesterol diet. This is observed, in particular, in 2008 among adults aged 20 years and older in the United States, when approximately 68% were overweight (of which, 33% were obese and 5% were extremely obese) [1]. Obesity is an important disease in the realm of preventive medicine because it is regarded as a risk factor associated with a broad spectrum of cardio-metabolic disturbances, including diabetes, hypertension, atherosclerosis and cardiovascular disease [2–5]. Several mechanisms induce the risk of obesity, including induced energy/high fat diet, increased intestinal adsorption, enhanced lipogenesis, suppressed lipolysis and fat oxidation and decreased energy expenditure. A high-fat diet has been shown to cause an elevation of plasma lipids, whereas a normal diet causes a decrease in the cholesterol levels in humans [6,7]. The two most common components of cholesterol are low-density lipoproteins (LDL) and high-density lipoproteins (HDL), and elevated serum levels of LDL cholesterol and reduced levels of HDL cholesterol are important independent risk factors for CVD [8,9]. Therefore, the control of postprandial LDL and HDL has been shown to be important in the treatment of CVD and the prevention of atherosclerosis.

Several publications have reported that the phytochemicals from natural products have a beneficial effect on CVD and atherosclerosis [10–13] by regulating multiple epigenetic mechanisms, including the release of HDL and LDL [14,15]. These reports have demonstrated a reduction in the incidence of CVD and atherosclerosis with obesity by natural product therapy.

The extracts of *Gastrodia* rhizome, a polysaccharide-enriched dry tuber of *Gastrodia elata* Blume (Orchidaceae), have been used in alternative traditional medicine in the Asian Pacific region as an anticonvulsant, an analgesic and a sedative against general paralysis, epilepsy, vertigo, and tetanus [16]. In addition to polysaccharides, including alpha-d-glucan, the major physiological compounds in *Gastrodia elata* Blume are gastrodin, parishin, and vanillyl alcohol, glycoprotein [17–19]. Among the bioactive components of *Gastrodia elata* Blume, the glycoproteins inhibit platelet aggregation and exhibit antithrombotic activity [17]. Moreover, we have been showed that acidic polysaccharide from *Gastrodia* rhizome has potential therapeutic ability of hypertension in SHR model [20]. Although *Gastrodia* rhizome has various biological actions [21,22], the effect of acidic polysaccharides purified from *Gastrodia* rhizome in SD rats fed a high-fat diet has not yet been studied by analysis of blood cholesterol including HDL and LDL to protect properties of CVD and atherosclerosis.

Several reports have been published on anti-atherosclerosis effect of acidic polysaccharide through the inhibition of nd presented the following: the polysaccharides fraction from red ginseng roots has a significant anti-cholesterol activity [23]. Ginseng has similar activities *Gastrodia* rhizome [24].

This study, therefore, investigated the anti-atherosclerotic potential of the acidic polysaccharides purified from *Gastrodia* rhizomes and evaluated the concentrations of serum lipids, including total cholesterol (TC), triglyceride (TG), HDL cholesterol, and LDL cholesterol, in SD rats that were fed a high-fat diet.

## 2. Results and Discussion

### 2.1. Body Weights and Food Intake

**Figure 1:**
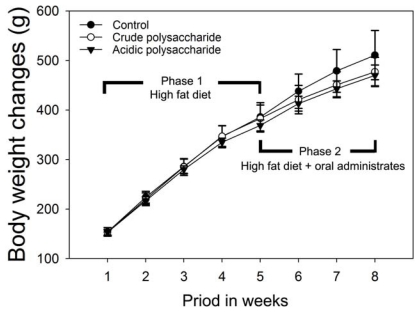
Effects of crude and acidic polysaccharides of *Gastrodia* rhizome on body weight changes during experiment periods. The body weight was recorded weekly during the 8 weeks experimental period. Values are expressed as the mean ± standard deviation.

There were no significant differences in the changes in the SD rat body weight (Figure 1), and there were no differences in the growth ratio among the control, crude and acidic polysaccharide groups prior to Phase 1 of the experiment (Figure 1). No animals died when housed at room temperature or subjected to the oral administration of the crude and acidic polysaccharides from *Gastrodia* rhizomes.

In Phase 2 of the experiment, there were no significant differences in the changes in the body weight, as in Phase 1. The food intake did not differ between the groups (control, crude polysaccharide and acidic polysaccharide as shown in Table 1). Interestingly, a tendency was observed for a reduction in the daily growth ratio among the crude polysaccharide (Group B, 3.81 ± 1.01%) and acidic polysaccharide (Group C, 3.91 ± 0.99%) groups compared with the control (Group A, 4.60 ± 1.51%) prior to the experiment (Figure 1). Park *et al*. [25] have been showed that *Gastrodia* rhizome treatment protects against insulin resistance by decreasing body fat by directly working on adipocytes. Consistent this finding, body weight was slightly reduced by crude and acidic polysaccharide *Gastrodia* rhizome in high fat diet-fed groups. These results indicated that crude and acidic polysaccharide from *Gastrodia* rhizome might be induced to protect against body fat accumulation by directly working on adipocyte development in obese.

Obesity is strongly related to metabolic disorders, such as CVD and atherosclerosis, which release total cholesterol [10,12]. Therefore, we examined whether the crude and acidic polysaccharides had inhibitory effects on the total cholesterol release that is induced by a high-fat diet.

### 2.2. Effects of the Crude and Acidic Polysaccharides of Gastrodia Rhizomes on the Total Cholesterol and Triglyceride Contents

**Figure 2:**
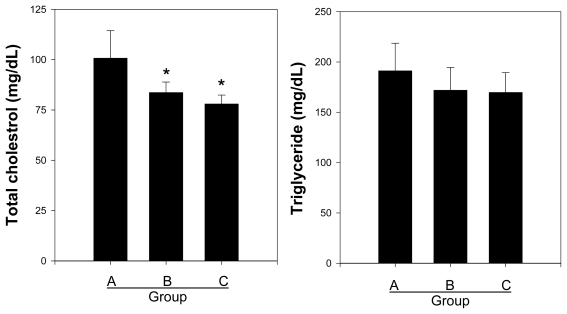
Effects of crude and acidic polysaccharide extracts of *Gastrodia* rhizomes on the total cholesterol and triglyceride contents. Each value given is the mean ± standard deviation of five individual plates and is representative of the results from at least five independent experiments. (Significance: * *P* < 0.05).

The total cholesterol and triglyceride levels were evaluated in the blood samples from the rats of each group. As shown in Figure 2, the crude polysaccharide (Group B, 83.62 ± 5.25 mg/dL) and acidic polysaccharide (Group C, 78.00 ± 4.42 mg/dL) groups demonstrated significantly decreased total cholesterol levels compared to the control (Group A, 100.7 ± 13.68 mg/dL). Interestingly, the acidic polysaccharides exhibited a greater reduction, by approximately 5%, in the total cholesterol induced by a high-fat diet, as compared with the crude polysaccharide extract. According to our previous results, the crude polysaccharide and acidic polysaccharide extracts from *Gastrodia* rhizome have different compositions [26]. Thus, we suggest that the above results were due to the different chemical compositions between crude and acidic polysaccharide extracts. However, there were no differences in the serum triglyceride levels among the control, crude polysaccharides and acidic polysaccharides. It was an interesting result that both the crude polysaccharide (Group B) and acidic polysaccharide (Group C) oral administrated groups suppressed total cholesterol on the high fat diet compared with control (Group A).

A correlation between the pathological findings seen on coronary angiography and cholesterol has been found in many studies [27]. Total cholesterol is composed of HDL and LDL cholesterol, and the serum levels of HDL and LDL are important in CVD and atherosclerosis [8,28]. A total cholesterol, high HDL or high LDL may be secondary to uncontrolled factors that promote cardiovascular disease in other ways and cause atherosclerosis at the same time, for instance lack of physical activity and obesity [29]. Hence, we investigated these levels among the treatment and control groups.

### 2.3. Effects of Crude and Acidic Polysaccharides of Gastrodia Rhizomes on HDL and LDL

The key principle in achieving weight loss in obese human is to modify lifestyle behaviors to decrease energy intake and increase physical activity. However, natural product derived polysaccharide therapy has limited long-term success, and many obese humans who lose weight regain their lost weight over time [30]. The failure to achieve permanent weight loss and the prevention of athersclerosis have led to increased efforts to determine a biological processed polysaccharide as an additional functional possibility in treating high HDL and regulation of LDL [6,31,32]. It is important to note that the development of atherosclerosis is prevalent in obese patient. Therefore, we investigated that the action of crude polysaccharide and acidic polysaccharide from the *Gastrodia* Rhizomes dampens the release of HDL and LDL cholesterol in blood that act on the regulating atherosclerosis *in vivo*.

**Figure 3:**
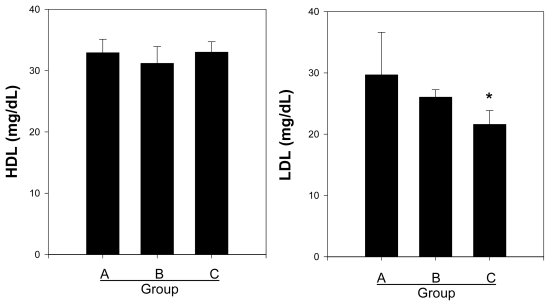
Effect of crude and acidic polysaccharides from *Gastrodia* rhizomes on high-density lipoproteins (HDL) and low-density lipoproteins (LDL) levels in blood serum. Each value given is the mean ± standard deviation of five individual plates and is representative of the results from at least five independent experiments. (Significance: * *P* < 0.05).

As shown in Figure 3, non-high fat diet groups showed no increased in the physiological concentration of serum HDL and LDL levels, whereas serum LDL cholesterol was higher in the control group, at 29.67 mg/dL, than in the treatment groups (Group B, 26.04 mg/dL and Group C, 21.06 mg/dL). In particular, the administration of the acidic polysaccharides significantly decreased the LDL cholesterol by 12.3% compared to the control group. However, there were no significant differences in the serum HDL cholesterol levels among the control (Group A, 32.92 ± 2.19 mg/dL) or the crude (Group B, 31.20 ± 2.73 mg/dL) or acidic polysaccharide (Group C, 33.01 ± 1.69 mg/dL) groups.

The averages from the analysis of the serum LDL cholesterol were very similar to those obtained for the total cholesterol, whereas the serum HDL cholesterol levels exhibited trends that were similar to those for the triglyceride levels.

### 2.4. Effects of Crude and Acidic Polysaccharides of Gastrodia Rhizomes on the Atherogenic Index

**Figure 4:**
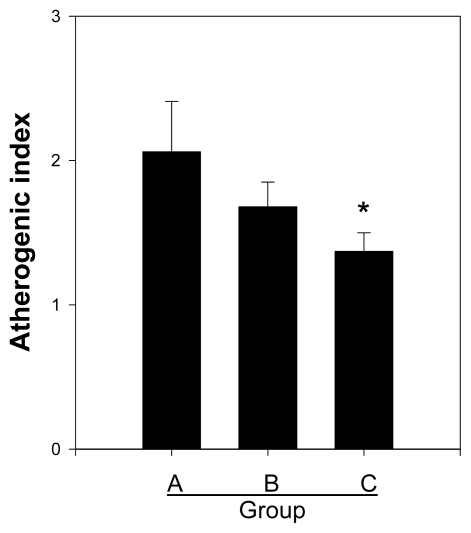
Effect of crude and acidic polysaccharides of *Gastrodia* rhizomes on the atherogenic index. Each value given is the mean ± standard deviation of five individual plates and is representative of the results from at least five independent experiments. (Significance: * *P* < 0.05).

To determine the effect of the crude and acidic polysaccharides on atherosclerosis in SD rats fed a high-fat diet, the atherogenic index was evaluated, as described in the experimental section. Figure 4 shows the average value of the atherogenic index of the control (Group A) and the crude polysaccharide (Group B) and acidic polysaccharide (Group C) treatment groups. The control (Group A, high fat diet) group demonstrated an index of 2.06 mg/dL, which was the highest value in our experiment, whereas the index for the crude polysaccharide group was 18.45% lower than the control, and the index of the acidic polysaccharide group was significantly decreased, by 32.95%, compared with the control. These results indicated that the acidic polysaccharide extract has an inhibitory effect on atherosclerosis in SD rats fed a high-fat diet.

## 3. Experimental Section

### 3.1. Materials

The extraction and purification of acidic polysaccharides from *Gastrodia* rhizomes were performed as described previously [20]. The acidic polysaccharide fractions, obtained by DEAE-Sepharose CL-6B, contained 0.32 ± 0.02% of total protein, 82.40 ± 1.16% of total sugar, and 17.28 ± 0.58% of acidic polysaccharides, respectively. The fraction of acidic polysaccharides consisted of xylose, glucose, galacturonic acid, and glucuronic acid.

### 3.2. Animal Experiments and Diet

Thirty male SD rats (4 weeks old) were purchased from Charles River Laboratories, Inc. (Yokohama, Japan) and used after 1 week of quarantine and acclimation. The animals were kept in the animal facility of the Korea Food Research Institute in a light-controlled room (12-h light/dark cycle) at an average temperature of 24 °C and relative humidity of 60%. This experiment was conducted in facilities approved by the Guiding Principles for the Care and Use of Laboratory Animals of the Ethics Committee of the Korea Food Research Institute. All of the rats were fed an AIN-93M–based diet supplemented with 10% lard, 2% corn oil, and 1% cholesterol as the source of the increased fat (Table 2). All of the rats were provided *ad libitum* access to the high-fat diet and tap water throughout the 8-week treatment period. After four weeks on the high-fat diet, the rats were randomly divided into three groups: the control group (A) fed only the high fat diet and the crude polysaccharide (B) and acidic polysaccharide (C) groups. As shown in Table 3, both the crude polysaccharide (6 mg/kg) and acidic polysaccharide (6 mg/kg) extracts were administered by oral gavage to the SD rats. The doses of the crude and acidic polysaccharides were determined by considering an effective dose of hot water and crude polysaccharides ranging from 3.6 to 14.7 mg/kg of body weight/day in the SD rats. The acidic polysaccharides consisted of a 12.56% fraction of the crude polysaccharide. The samples were administered by oral gavage at 1 mL/250 g of body weight on a daily basis, 7 days per week for 5 weeks. The control animals were treated with the same volume of distilled water.

### 3.3. Body Weight, Food Intake and Water Consumption

The body weights were measured at the initiation of the treatment, every week thereafter, and on the day of sacrifice. The food intake was measured in g/kg bw/day at the start of the treatment and at weekly intervals thereafter. The amount of food was measured before being supplied to the cage; any food that remained the following day was also weighed.

### 3.4. Blood Chemistry

At the end of the treatment, all of the rats were fasted for 12 h, anesthetized with isoflurane and euthanized; arteriovenous blood was collected. The blood samples were evaluated for serum lipid levels and atherogenic index. The concentrations of TC, TG, HDL cholesterol and LDL cholesterol were determined using a commercial kit (Asan Pharmaceutical Co., Seoul, Korea). The atherogenic index (AI) was calculated using the following formula: (TC-HDL)/HDL.

### 3.5. Statistical Analysis

The data are presented as the means ± standard deviation. The data were analyzed by one-way analysis of variance (ANOVA), and the all of the grouped data were evaluated by one-way analysis of variance, followed by the least significant difference test using SPSS [33]. Values of *P* < 0.05 were considered significantly different between treatments.

## 4. Conclusions

This article describes the effects of oral administration of crude and acidic polysaccharides (PS) from Gastrodia rhizomes along with a HFD to rats on serum lipid levels. It is interesting and surprising that this administration resulted in a significant decrease in circulating cholesterol, mainly from LDL. This type of finding makes one question the mechanism of action. If the authors offered at least some mechanism either *in vivo* or *in vitro*, it would make this study much more interesting. In this study, we provided the first description of a positive and direct effect of crude and acidic polysaccharide extracts from *Gastrodia* rhizomes for the serum cholesterol, HDL, LDL and atherosclerotic risks in SD rats. Here, we demonstrated that the crude and acidic polysaccharide groups exhibited decreased levels of total serum cholesterol. Furthermore, the change in the daily body weight tended to decrease in the crude and acidic polysaccharide groups. Interestingly, our data suggested that the acidic polysaccharide significantly decreased the LDL levels and atherogenic index, as compared with the control group. Taken together, we demonstrated that acidic polysaccharide extracts from *Gastrodia* rhizomes dramatically suppress the atherosclerosis risk by reducing the total serum cholesterol and LDL levels in SD rats. Thus, we conclude that the acidic polysaccharide extract from *Gastrodia* rhizomes might have beneficial effects in lowering the incidence of CVD and atherosclerosis by reducing the *de novo* synthesis of total cholesterol and LDL.

## Figures and Tables

**Table 1:** Mean food intake of SD rats fed a high-fat diet for 4 weeks, comprising Phase 1 of the experiment. Group A, the control was given a high-fat diet with distilled water; Group B was given a high-fat diet plus the crude polysaccharides of *Gastrodia* rhizomes; Group C was given a high-fat diet with the acidic polysaccharides of *Gastrodia* rhizomes. The final 4 weeks of the experiment comprised Phase 2.

Group (*n* = 10)	Food Intake (g/weeks)							

	1	2	3	4	5	6	7	8
**A**	14.64 ± 0.50	22.26 ± 1.29	22.58 ± 1.57	24.59 ± 2.21	26.22 ± 1.83	27.15 ± 2.08	28.96 ± 2.94	25.46 ± 2.25
**B**	14.57 ± 0.53	21.22 ± 1.76	21.51 ± 1.36	23.43 ± 1.54	25.72 ± 0.82	25.30 ± 1.32	26.87 ± 1.62	26.23 ± 6.23
**C**	14.76 ± 0.28	20.82 ± 1.17	23.20 ± 3.11	24.41 ± 1.39	24.04 ± 1.24	25.28 ± 1.24	25.37 ± 1.81	27.41 ± 1.29

**Table 2:** Composition of the experimental diet based on the AIN-93 diet with high fat.

Ingredients	Content (%)
Casein (feed grade CP 85%)	20.00
Corn starch	39.75
Dextrinized corn starch	13.20
Sucrose	10.00
Soybean oil	7.00
Cellulose (fiber)	5.00
Mineral mixture 1	3.50
Vitamin mixture 2	1.00
l-Cysteine	0.30
Choline bitartrate	0.25

1 Contained per kg mixture: 500 g CaHPO_4_, 74 g NaCl, 220 g K_3_C_6_O_7_·H_2_O, 52 g K_2_SO_4_, 24 g MgO, 3.5 g Mn (48%), 6.0 g Fe (17%), 1.6 g Zn (70%), 0.3 g Cu (53%), 0.01 g KIO_3_, 0.55 g CrK(SO_4_)_2_·12H_2_O and sucrose;

2 Contained per kg mixture: 600 mg thiamine·HCl, 600 mg riboflavin, 700 mg pyridoxine·HCl, 3 g nicotinic acid, 400,000 IU vitamin A (retinyl acetate), 5000 IU vitamin E (dl-α-tocopheryl acetate), 2.5 mg vitamin D_3_, 5.0 mg vitamin K and sucrose.

**Table 3:** Experimental design.

Group (*n* = 12)	Phase 1 (4 weeks)	Phase 2 (4 weeks)
A^1^	HFD^2^	HFD
B	HFD	HFD + Crude polysaccharides
C	HFD	HFD + Acidic polysaccharides

1 Group A was orally administered with the same volume of distilled water, Groups B and C were orally administered crude and acidic polysaccharide extracts, respectively, of *Gastrodia* rhizomes at a concentration of 6 mg/kg using a stainless-steel oral tube for 5 weeks;

2 HFD (high-fat diet): the AIN diet-based commercial rat chow containing 10% lard, 2% corn oil, and 1% cholesterol (w/w).

## References

[1] Flegal K.M., Carroll M.D., Ogden C.L., Curtin L.R. (2010). Prevalence and trends in obesity among US adults, 1999–2008. JAMA.

[2] Larsson B., Svardsudd K., Welin L., Wilhelmsen L., Bjorntorp P., Tibblin G. (1984). Abdominal adipose tissue distribution, obesity, and risk of cardiovascular disease and death: 13 year follow up of participants in the study of men born in 1913. Br. Med. J. (Clin. Res. Ed.).

[3] Kahn S.E., Hull R.L., Utzschneider K.M. (2006). Mechanisms linking obesity to insulin resistance and type 2 diabetes. Nature.

[4] Purkayastha S., Zhang G., Cai D. (2011). Uncoupling the mechanisms of obesity and hypertension by targeting hypothalamic IKK-β and NF-κB. Nat. Med.

[5] Rocha V.Z., Libby P. (2009). Obesity, inflammation, and atherosclerosis. Nat. Rev. Cardiol.

[6] Beveridge J.M., Connell W.F., Mayer G.A., Firstbrook J.B., Dewolfe M.S. (1955). The effects of certain vegetable and animal fats on the plasma lipids of humans. J. Nutr.

[7] Ahrens E.H., Insull W., Blomstrand R., Hirsch J., Tsaltas T.T., Peterson M.L. (1957). The influence of dietary fats on serum-lipid levels in man. Lancet.

[8] Ballantyne C.M., Olsson A.G., Cook T.J., Mercuri M.F., Pedersen T.R., Kjekshus J. (2001). Influence of low high-density lipoprotein cholesterol and elevated triglyceride on coronary heart disease events and response to simvastatin therapy in 4S. Circulation.

[9] Obarzanek E., Sacks F.M., Vollmer W.M., Bray G.A., Miller E.R., Lin P.H., Karanja N.M., Most-Windhauser M.M., Moore T.J., Swain J.F. (2001). Effects on blood lipids of a blood pressure-lowering diet: the dietary approaches to stop hypertension (dash) trial. Am. J. Clin. Nutr.

[10] Cassidy A., O’Reilly E.J., Kay C., Sampson L., Franz M., Forman J.P., Curhan G., Rimm E.B. (2011). Habitual intake of flavonoid subclasses and incident hypertension in adults. Am. J. Clin. Nutr.

[11] Dohadwala M.M., Hamburg N.M., Holbrook M., Kim B.H., Duess M.A., Levit A., Titas M., Chung W.B., Vincent F.B., Caiano T.L. (2011). Effects of Concord grape juice on ambulatory blood pressure in prehypertension and stage 1 hypertension. Am. J. Clin. Nutr.

[12] Hooper L., Kroon P.A., Rimm E.B., Cohn J.S., Harvey I., le Cornu K.A., Ryder J.J., Hall W.L., Cassidy A. (2008). Flavonoids, flavonoid-rich foods, and cardiovascular risk: A meta-analysis of randomized controlled trials. Am. J. Clin. Nutr.

[13] Chen C.C., Hsu J.D., Wang S.F., Chiang H.C., Yang M.Y., Kao E.S., Ho Y.C., Wang C.J. (2003). *Hibiscus sabdariffa* extract inhibits the development of atherosclerosis in cholesterol-fed rabbits. J. Agric. Food. Chem.

[14] Lin W.Y., Chiu T.Y., Lee L.T., Lin C.C., Huang C.Y., Huang K.C. (2008). Betel nut chewing is associated with increased risk of cardiovascular disease and all-cause mortality in Taiwanese men. Am. J. Clin. Nutr.

[15] Al-Awwadi N.A., Araiz C., Bornet A., Delbosc S., Cristol J.P., Linck N., Azay J., Teissedre P.L., Cros G. (2005). Extracts enriched in different polyphenolic families normalize increased cardiac NADPH oxidase expression while having differential effects on insulin resistance, hypertension, and cardiac hypertrophy in high-fructose-fed rats. J. Agric. Food. Chem.

[16] Tang W., Eisenbrand G (1992). Chinese Drugs of Plant Origin. Chemistry, Pharmacology, and Use in Traditional and Modern Medicine.

[17] Ding C.S., Shen Y.S., Li G., Wei Z., Wei F. (2007). Study of a glycoprotein from *Gastrodia elata*: Its effects of anticoagulation and antithrombosis. Zhong Guo Zhong Yao Za Zhi.

[18] Gutiiérrez R.M.P. (2010). Orchids: A review of uses in traditional medicine, its phytochemistry and pharmacology. J. Med. Plants Res.

[19] Lu W.B., Zhang B.E., Wang J., Lu R.X., Li R.L., Chen W.W. (2010). Screening of anti-diarrhea effective fractions from guava leaf. Zhong Yao Cai.

[20] Lee O.H., Kim K.I., Han C.K., Kim Y.C., Hong H.D. (2012). Effects of acidic polysaccharides from *Gastrodia* rhizome on systolic blood pressure and serum lipid concentrations in spontaneously hypertensive rats fed a high-fat diet. Int. J. Mol. Sci.

[21] Kim H.J., Moon K.D., Lee D.S., Lee S.H. (2003). Ethyl ether fraction of *Gastrodia elata* Blume protects amyloid β peptide-induced cell death. J. Ethnopharmacol.

[22] Xu X., Lu Y., Bie X. (2007). Protective effects of gastrodin on hypoxia-induced toxicity in primary cultures of rat cortical neurons. Planta Med.

[23] Kwak Y.S., Kyung J.S., Kim J.S., Cho J.Y., Rhee M.H. (2010). Anti-hyperlipidemic effects of red ginseng acidic polysaccharide from Korean red ginseng. Biol. Pharm. Bull.

[24] Zhou J. (1991). Bioactive glycosides from Chinese medicines. Mem. Inst. Oswaldo Cruz.

[25] Park S., Kim D.S., Kang S. (2011). *Gastrodia elata* Blume water extracts improve insulin resistance by decreasing body fat in diet-induced obese rats: Vanillin and 4-hydroxybenzaldehyde are the bioactive candidates. Eur. J. Nutr.

[26] Hong H.D., Shin E.J., Kim K.I., Choi S.Y., Han C.K. (2007). Effect of *Gastrodia elata* Blume components on systolic blood pressure and serum lipid concentrations in spontaneously hypertensive rats fed high fat diet. J. Korean Soc. Food Sci. Nutr.

[27] Pearson T.A. (1984). Coronary arteriography in the study of the epidemiology of coronary artery disease. Epidemiol. Rev.

[28] Armstrong V.W., Cremer P., Eberle E., Manke A., Schulze F., Wieland H., Kreuzer H., Seidel D. (1986). The association between serum Lp(a) concentrations and angiographically assessed coronary atherosclerosis. Dependence on serum LDL levels. Atherosclerosis.

[29] Ashen M.D., Blumenthal R.S. (2005). Low HDL cholesterol levels. New Engl. J. Med.

[30] Pittler M.H., Ernst E. (2004). Dietary supplements for body-weight reduction: A systematic review. Am. J. Clin. Nutr.

[31] Hsu C.H., Tsai T.H., Kao Y.H., Hwang K.C., Tseng T.Y., Chou P. (2008). Effect of green tea extract on obese women: A randomized, double-blind, placebo-controlled clinical trial. Clin. Nutr.

[32] Devi K.S., Kurup P.A. (1972). Hypolipidaemic activity of *Phaseolus mungo* (blackgram) in rats fed a high-fat-high-cholesterol diet. Isolation of a protein and polysaccharide fraction. Atherosclerosis.

[33] (2005). *SPSS*, version 10; the statistical software SPSS for windows.

